# Identification of immunodiagnostic blood biomarkers associated with spinal cord injury severity

**DOI:** 10.3389/fimmu.2023.1101564

**Published:** 2023-03-29

**Authors:** Jianfeng Li, Xizhe Liu, Jianmin Wang, Fuan Wang, Zhengya Zhu, Tao Tang, Jun Wang, Zhiyu Zhou, Manman Gao, Shaoyu Liu

**Affiliations:** ^1^ Innovation Platform of Regeneration and Repair of Spinal Cord and Nerve Injury, Department of Orthopaedic Surgery, The Seventh Affiliated Hospital, Sun Yat-sen University, Shenzhen, Guangdong, China; ^2^ Guangdong Provincial Key Laboratory of Orthopedics and Traumatology, Orthopaedic Research Institute/Department of Spinal Surgery, The First Affiliated Hospital of Sun Yat-sen University, Guangzhou, Guangdong, China; ^3^ Department of Sport Medicine, Inst Translat Med, The First Affiliated Hospital of Shenzhen University, Shenzhen Second People’s Hospital, Shenzhen, Guangdong, China; ^4^ Guangdong Key Laboratory for Biomedical Measurements and Ultrasound Imaging, School of Biomedical Engineering, Shenzhen University Health Science Center, Shenzhen, Guangdong, China

**Keywords:** spinal cord injury, blood, immune cells, immune-related genes, biomarkers, diagnosis

## Abstract

Blood always shows some immune changes after spinal cord injury (SCI), and detection of such changes in blood may be helpful for diagnosis and treatment of SCI. However, studies to date on blood immune changes after SCI in humans are not comprehensive. Therefore, to obtain the characteristics of blood immune changes and immunodiagnostic blood biomarkers of SCI and its different grades, a human blood transcriptome sequencing dataset was downloaded and analyzed to obtain differentially expressed immune-related genes (DEIGs), related functions and signaling pathways related to SCI and its various grades. Characteristic biomarkers of SCI and its different grades were identified by using weighted gene coexpression network analysis (WGCNA) and least absolute shrinkage and selection operator (LASSO) logistic regression. Expression of biomarkers was verified through experiments. The area under the curve (AUC) of biomarkers was calculated to evaluate their diagnostic value, and differences in immune cell content were examined. In this study, 17 kinds of immune cells with different contents between the SCI group and healthy control (HC) group were identified, with 7 immune cell types being significantly increased. Differences in the content of immune cells between different grades of SCI and the HC group were also discovered. DEIGs were identified, with alteration in some immune-related signaling pathways, vascular endothelial growth factor signaling pathways, and axon guidance signaling pathways. The SCI biomarkers identified and those of American Spinal Injury Society Impairment Scale (AIS) A and AIS D of SCI have certain diagnostic sensitivity. Analysis of the correlation of immune cells and biomarkers showed that biomarkers of SCI, AIS A grade and AIS D grade correlated positively or negatively with some immune cells. CKLF, EDNRB, FCER1G, SORT1, and TNFSF13B can be used as immune biomarkers for SCI. Additionally, GDF11and HSPA1L can be used as biomarkers of SCI AIS A grade; PRKCA and CMTM2 can be used as biomarkers of the SCI AIS D grade. Detecting expression of these putative biomarkers and changes in related immune cells may be helpful for predicting the severity of SCI.

## 1 Introduction

SCI is an important central nervous system disorder and cause of limb paralysis (1). The global incidence of SCI has increased from 236 to 1298 cases per million population over the past 30 years. The annual global incidence of SCI is estimated to be between 250,000 and 500,000 people (2). SCI is divided into primary injury and secondary injury. Primary injury refers to mechanical injury caused by an external force on the spinal cord (3, 4); secondary damage is caused by a series of cascade reactions, such as inflammation, ischemia, electrolyte imbalance, and oxidative stress, after spinal cord injury (4, 5). The immune response plays an important role in secondary injury of the spinal cord and can not only aggravate the injury but also promote repair of the spinal cord (5, 6). After SCI, hemorrhage and edema of the gray matter and white matter in the spinal cord tissues occur to varying degrees; local arterial blood supply disorders and obstruction of venous return can lead to death and disintegration of nerve cells in a short time, and biomarkers can enter the blood circulation through the broken blood-spinal barrier (7, 8). In turn, with secondary SCI, many immune cells and cytokines in the blood enter the injured spinal cord area through the blood-brain barrier, playing an important role in the local microenvironmental immune response (9, 10). For example, blood-derived M1 and M2 macrophages, as well as neutrophils and lymphocytes, have a role in secondary SCI (11–17). Furthermore, the immune state of blood is altered to a certain extent, reflecting the degree of SCI (18–20). Therefore, the search for biomarkers of blood immune characteristics in patients with different grades of SCI may be helpful for auxiliary diagnosis and treatment of SCI.

SCI is divided into five grades from severe to mild according to classification of the functional status of SCI by the American Spinal Cord Injury Association Impairment Scale (AIS), AIS A, AIS B, AIS C, AIS D and AIS E, with the sensation and function of AIS E grade being completely normal (21, 22). At present, the grading and severity of SCI are usually determined clinically based on magnetic resonance imaging (MRI) and physical examination by specialists (23–25). However, MRI is not always available. Moreover, it is difficult to accurately determine the degree of SCI in the acute phase of SCI and when the patient is unresponsive, and it is contraindicated in some patients, such as those with penetrating metal injuries; it is also inconvenient to transport patients with SCI for MRI examination (26, 27). Additionally, it is impossible to obtain tissue samples of SCI to assess the condition. In general, detection of biomarkers in blood may compensate for the limitations of imaging diagnosis.

**Figure 1:**
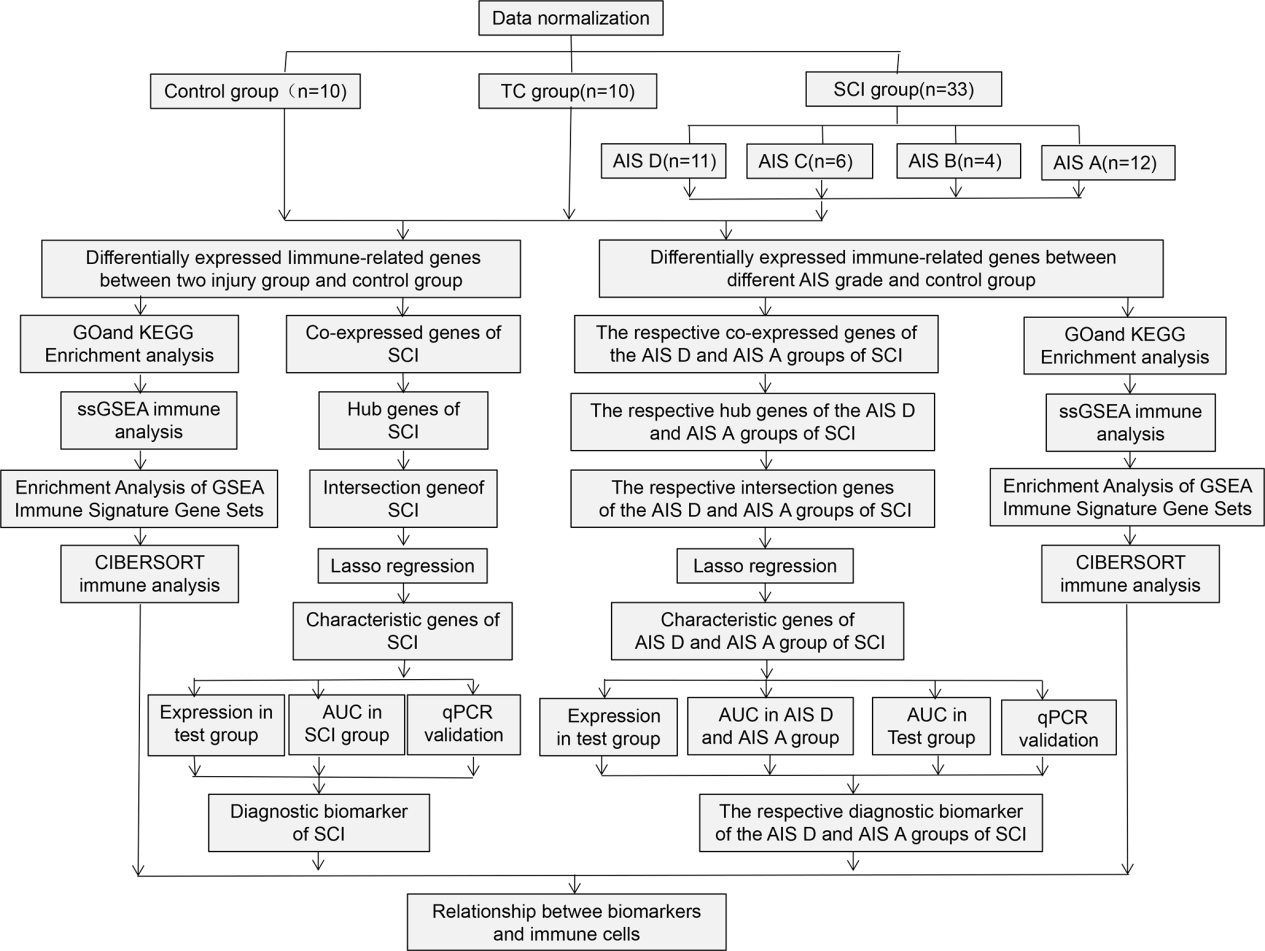
Flowchart of this research work.

Despite many studies on blood biomarkers for SCI, research to date is not comprehensive. Studies on blood immunodiagnostic biomarkers for SCI are also incomplete. Therefore, to identify the blood immunodiagnostic biomarkers and the characteristics of blood immune changes in SCI and its different grades, this study systematically investigated changes in peripheral blood immune cells and differential expression of immune-related genes after SCI and identified immunodiagnostic biomarkers for SCI and for AIS A and AIS D grades, providing an experimental basis for diagnosis and treatment of spinal cord injury. The workflow of this study is shown in **Figure 1**.

## 2 Materials and methods

### 2.1 Data source and normalization

Due to the lack of data on human SCI blood, we searched multiple databases but were only able to retrieve a significant transcriptome sequencing dataset GSE151371 from the gene expression omnibus (GEO) database. The sequencing platform of this dataset is GPL20301 Illumina HiSeq 4000 (Homo sapiens). The dataset contains blood samples from 10 healthy uninjured controls (HC group), 10 blood samples from trauma controls with noncentral nervous system injury (TC group), and 38 blood samples from SCI patients (SCI group). The SCI patients with complete follow-up data were divided into four grades, AIS A, AIS B, AIS C, and AIS D, according to the AIS. Among them, there were 12 patients with grade AIS A, 4 patients with grade AIS B, 6 patients with grade AIS C, and 11 patients with grade AIS D. Blood sampling in all SCI patients was performed within hours after SCI (30.3 ± 18.9 hours). Then, we corrected the individual microarray data separately and obtained a list of human immunity-related genes from the ImmPort (https://www.immport.org/home) website.

### 2.2 Identification of differentially expressed genes

The Bioconductor “limma” package was used to obtain differentially expressed genes (DEGs) and DEIGs between blood samples from patients with SCI and blood samples from TCs and HCs, which were visualized using the “ggplot2” package (28). We extracted the DEIGs from the DEGs according to the downloaded immune gene list.

### 2.3 GO, KEGG and GSEA enrichment analyses

Gene Ontology (GO) analysis and Kyoto Encyclopedia of Genes and Genomes (KEGG) pathway enrichment analysis of DEGs and DEIGs were performed by the “org.Hs.eg.db”, “clusterProfiler”, “ggplot2” and “enrichplot” packages (29), and a p value < 0.05 was set as the cutoff criterion. Gene set enrichment analysis (GSEA) of immune-related genes was performed by the “org.Hs.eg.db”, “clusterProfiler”, “limma” and “enrichplot” packages, and a p value < 0.05 was set as the cutoff criterion. When we drew the figure, a q-value <0.05 was set as the cutoff criterion.

### 2.4 Identification of diagnostic signature genes for spinal cord injury

WGCNA was used to analyze and identify the module with the highest correlation coefficient for disease characteristics in SCI and AIS A and AIS D grades and to obtain important genes related to this module. LASSO logistic regression was applied to identify characteristically expressed immune-related genes of SCI and its different grades based on hub genes (30). Experimental datasets were used to determine the potential predictive value of diagnostic signature genes for SCI and for grading its severity. Diagnostic value was determined by the AUC of the receiver operating characteristic (ROC) curve. P<0.05 was considered statistically significant.

### 2.5 Immune cell assessment

The status of immune cells in the blood of SCI patients and those with different grades was assessed using single-sample gene set enrichment analysis (ssGSEA) and Cell-type Identification by Estimating Relative Subsets of RNA Transcripts (CIBERSORT) (31, 32). ssGSEA converted the gene expression profile of a single sample into a gene set enrichment profile, enabling us to describe the cell state according to the activity level of biological processes and pathways and to calculate the immune cell infiltration score according to the gene set related to immune cell markers (31). The CIBERSORT algorithm was used to estimate the abundance of immune cells by deconvolving the expression matrix of immune cell subtypes according to the principle of linear support vector regression (32). Additionally, the relationship between important hub genes and immune cells was assessed and plotted using R packages “limma”, “tidyverse”, “ggplot2”, “ggExtra”, “vioplot”, “reshape2”, and “ggpubr”.

### 2.6 Quantitative polymerase chain reaction(qPCR) for biomarker expression

This study was reviewed and approved by the ethics committee of the Seventh Affiliated Hospital of Sun Yat-sen University and was adhered to the tenets of the Declaration of Helsinki. All participants provided written informed consent before they were included in the study. Blood samples were collected from 10 patients with SCI, 3 patients with closed noncentral nervous system trauma, and 3 healthy individuals. According to a method described in the literature, ACK Lysing Buffer (Thermo Fisher Scientific, Waltham, MA, USA) was used to lyse red blood cells to obtain white blood cells in blood (33). The RNA of these white cells was extracted using an RNA extraction kit (Beyotime, Shanghai, China). The RNA was reverse‐transcribed to cDNA using PrimeScript RT Master Mix (TAKARA, Dalian, China), and expression levels of the biomarkers of SCI and different ASI grades were verified by qPCR. PowerUp SYBR reagents (Thermo Fisher Scientific, Waltham, MA, USA) and the qPCR platform from Bio‐Rad (Hercules, CA, USA) were used. The primer sequences used for qPCR were listed in **Table 1**.

**Table 1:** Primers used for RT-qPCR.

Gene	Sequences (5’–3’)
GAPDH	Forward-TGTGGGCATCAATGGATTTGG
	Reverse-ACACCATGTATTCCGGGTCAAT
CKLF	Forward-GCTGCGGCTGGATATTATCAA
	Reverse-CCAACACAGATACGATGAGCAT
EDNRB	Forward-TGCTGGGGATCATCGGGAA
	Reverse-GCGATCAAGATATTGGGACCGT
FCER1G	Forward-AGCAGTGGTCTTGCTCTTACT
	Reverse-TGCCTTTCGCACTTGGATCTT
SORT1	Forward-AAGTCTTTGGACCGACATCTCT
	Reverse-AGCACGCTTGTTATGTAGACG
TNFSF13B	Forward-GGGAGCAGTCACGCCTTAC
	Reverse-GATCGGACAGAGGGGCTTT
GDF11	Forward-CCACCGAGACCGTCATTAGC
	Reverse-CAGGCCGTAGGTACACCCA
HSPA1L	Forward-TAAACGTCTGATCGGCAGGAA
	Reverse-GCACGGTAATCACTGCATTGG
TNFRSF25	Forward-CCGTCCAGTTGGTGGGTAAC
	Reverse-CCATCACGTCGTAGAGCTGC
PRKCA	Forward-GTCCACAAGAGGTGCCATGAA
	Reverse-AAGGTGGGGCTTCCGTAAGT
CMTM2	Forward-AAGAAGGACGGTAAGGAGCCA
	Reverse-GCACCGCCTTTTGAGGTTTG

### 2.7 Statistical analysis

The qPCR data are expressed as mean ± SEM from at least three independent experiments. Student’s t‐test was used for two‐group comparison of qPCR data. P < 0.05 was considered significant.

## 3 Results

**Figure 2:**
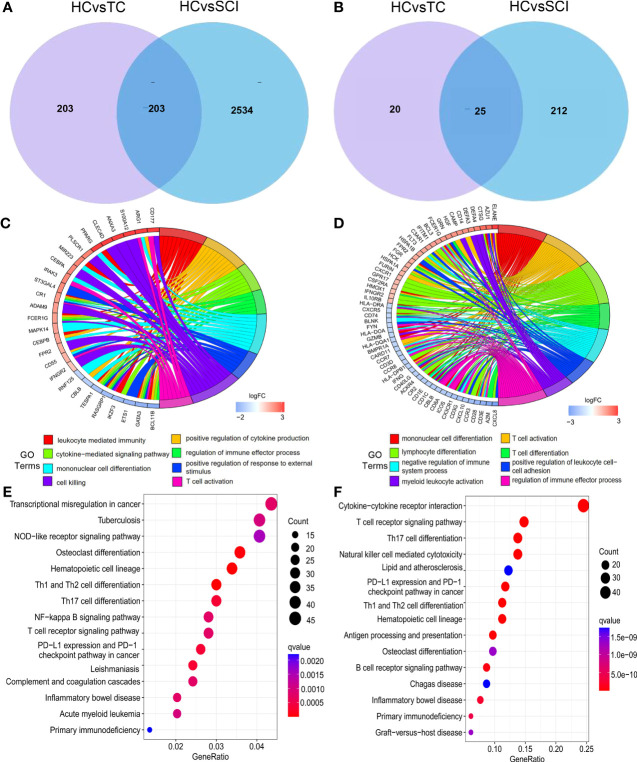
DEGs and their enrichment analysis. **(A)** Venn diagram of DEGs between the TC group and HC group and DEGs between the SCI group and HC group. **(B)** Venn diagram of DEIGs between the TC group and HC group and DEIGs between the SCI group and HC group. **(C)** GO functional analysis of DEGs between the SCI group and HC group. **(D)** GO functional analysis of DEIGs between the SCI group and HC group. **(E)** KEGG enrichment analysis of DEGs between the SCI group and HC group. **(F)** KEGG enrichment analysis of DEIGs between the SCI group and HC group. SCI, spinal cord injury; HC, healthy uninjured control; TC, trauma controls with noncentral nervous system injury; DEGs, differentially expressed genes; DEIGs, differentially expressed immune-related genes; GO, Gene Ontology; KEGG, Kyoto Encyclopedia of Genes and Genomes.

A total of 406 DEGs between the TC group and HC group, 2737 DEGs between the SCI group and HC group, and 237 DEIGs between the SCI group and HC group were identified (**Figures 2A, B**; **Supplement Table 1**). In addition, 2534 DEGs and 212 DEIGs specific to the SCI group relative to the TC group were identified (**Figures 2A, B**; **Supplement Table 2**). GO functional enrichment analysis of the DEGs between the SCI group and HC group showed that the functions of these genes are mainly related to leukocyte-mediated immune response, activation and differentiation of myeloid leukocytes, adhesion between leukocytes (**Figure 2C**; **Supplement Table 3**). Based on GO function enrichment analysis of the DEIGs between the SCI group and HC group, the functions of these genes are involved in regulation of cytokine production, leukocyte-mediated immune response, activation and differentiation of myeloid leukocytes, and regulation of response to external stimulus, among others (**Figure 2D**; **Supplement Table 4**). KEGG enrichment analysis of the DEGs between the SCI group and HC group showed that lymphocyte differentiation, the NF-kappa B signaling pathway, apoptosis and other signaling pathways were altered (**Figure 2E**; **Supplement Table 5**); cytokine−cytokine receptor interactions, T-cell receptor signaling, Th1, Th2 and Th17-cell differentiation, natural killer cell-mediated cytotoxicity, antigen processing and presentation, B-cell receptor signaling, NF-κB signaling pathway changes, JAK-STAT signaling pathway, Toll-like receptor signaling pathway, chemokine signaling pathway, vascular endothelial growth factor signaling pathway, axon guidance, leukocyte trans endothelial migration and other signaling pathways were changed between the SCI group and HC group (**Figure 2F**; **Supplement Table 6**).

**Figure 3:**
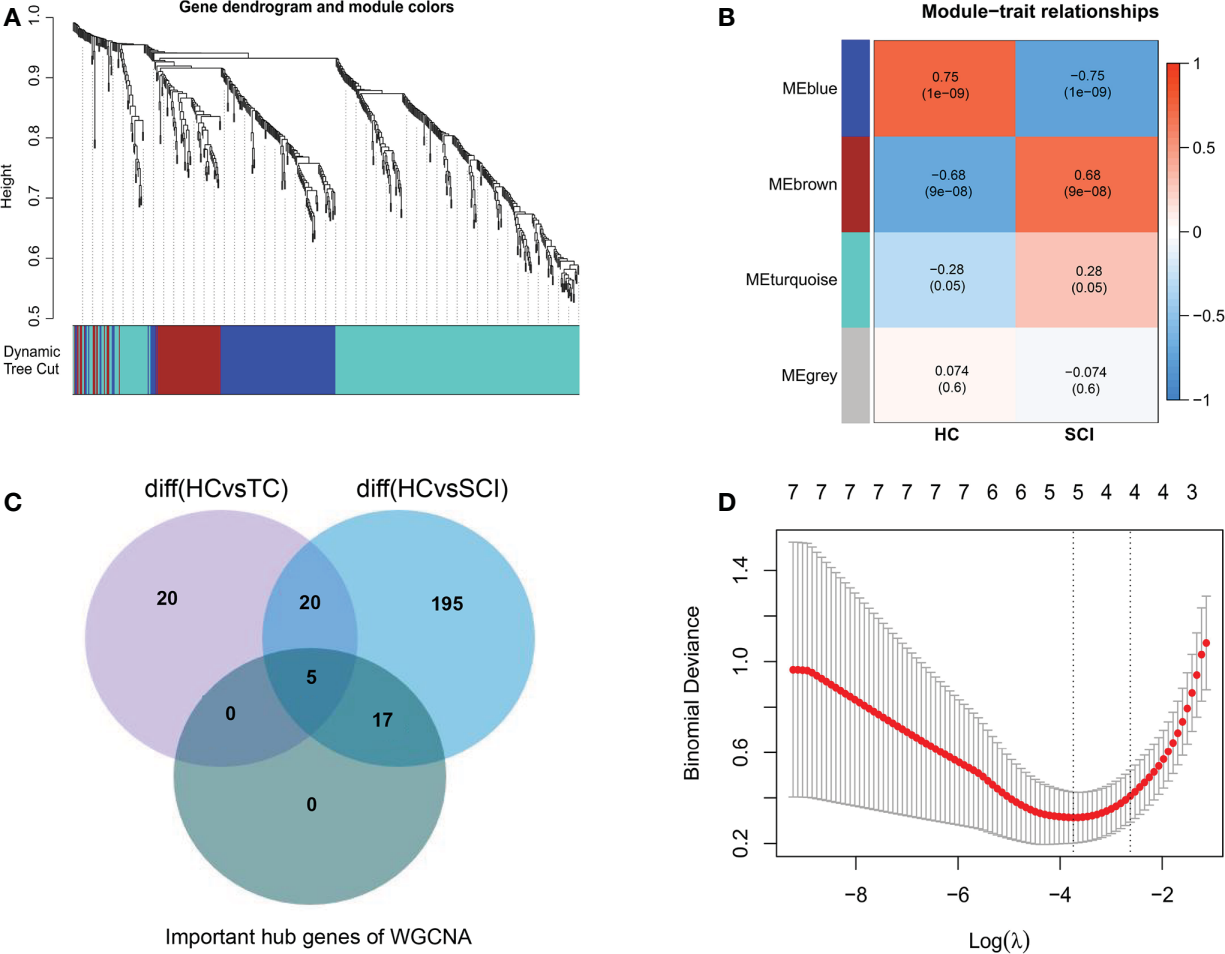
Identification of characteristic biomarkers of SCI. **(A)** Dendrogram of immune-related genes in SCI. Each branch of the figure represents one gene, and every color below represents a coexpression module. **(B)** Heatmap of the correlations between module signature genes and clinical features of SCI. Each row represents a module trait gene, and each column represents a trait. Each cell includes the corresponding correlation and p value. **(C)** WGCNA hub immune-related genes, DEIGs in SCI, and DEIGs in the TC Venn diagram. **(D)** LASSO logistic regression analysis. A P value < 0.05 was regarded as statistically significant. SCI, spinal cord injury; HC, healthy uninjured control; TC, trauma control with noncentral nervous system injury; WGCNA, weighted gene coexpression network analysis; AUC, area under the curve; DEIGs, differentially expressed immune-related genes.

**Figure 4:**
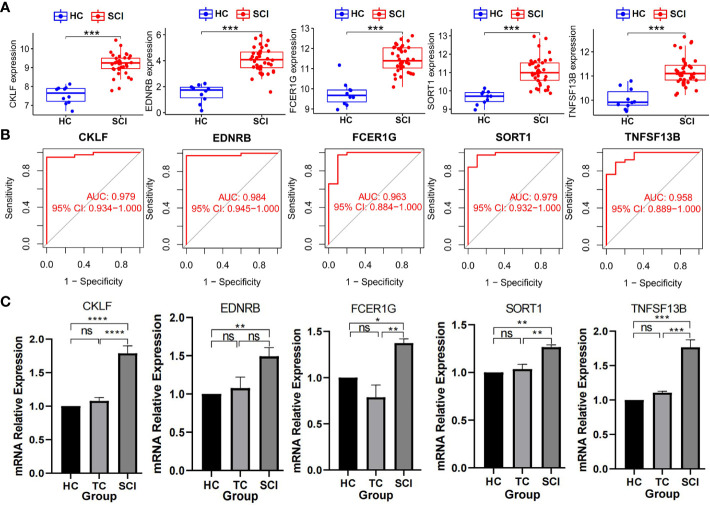
Detection of biomarkers of SCI. **(A)** Expression of biomarkers between SCI group and HC group in data set. ***P < 0.005; A P value < 0.5 was regarded as statistically significant. **(B)** AUC values of the biomarkers. **(C)** Expression levels of biomarkers detected by qPCR. Values are expressed as the means ± SEM. n = 3 for each group. Student’s t-test was used. ****P < 0.0001; ***P < 0.001; **P < 0.01; *P < 0.05; ns, no significance. A P value < 0.05 was regarded as statistically significant. SCI, spinal cord injury; HC, healthy uninjured control; TC, trauma control with noncentral nervous system injury; AUC, area under the curve; qPCR, quantitative polymerase chain reaction.

Subsequently, by performing WGCNA of expression of immune-related genes in SCI, 4 distinct coexpression modules were generated, and the correlation between each module and SCI was determined (**Figures 3A, B**). Based on the WGCNA heatmap, the MEbrown module correlated highly positively with SCI, and 90 shared immune-related genes of this module and 22 hub genes among the shared genes were obtained. Then, we determined the intersection of these hub genes with the DEIGs between the TC group and HC group and the DEIGs between the SCI group and HC group, and 17 immune-related genes only in the intersection between the hub genes and the DEIGs between the SCI group and HC group were identified (**Figure 3C**). Five important characteristic genes, CKLF, EDNRB, FCER1G, SORT1, and TNFSF13B, were obtained by analyzing expression of the 17 genes in these samples using LASSO logistic regression (**Figure 3D**). Expression of these characteristic biomarkers was detected in the dataset composed of the SCI group and HC group, with the expression levels of these five characteristic biomarkers being significantly upregulated in the SCI group (**Figure 4A**). Subsequently, the AUCs of these characteristic biomarkers in the dataset composed of the SCI group and HC group were calculated, at 0.979, 0.964, 0.963, 0.979 and 0.958 for CKLF, EDNRB, FCER1G, SORT1, and TNFSF13B, respectively (**Figure 4B**). Moreover, qPCR was used to detect the expression status of these biomarkers in blood samples of healthy adults and patients with SCI, and it was found that expression of these biomarkers was significantly upregulated in the SCI group (**Figure 4C**).

**Figure 5:**
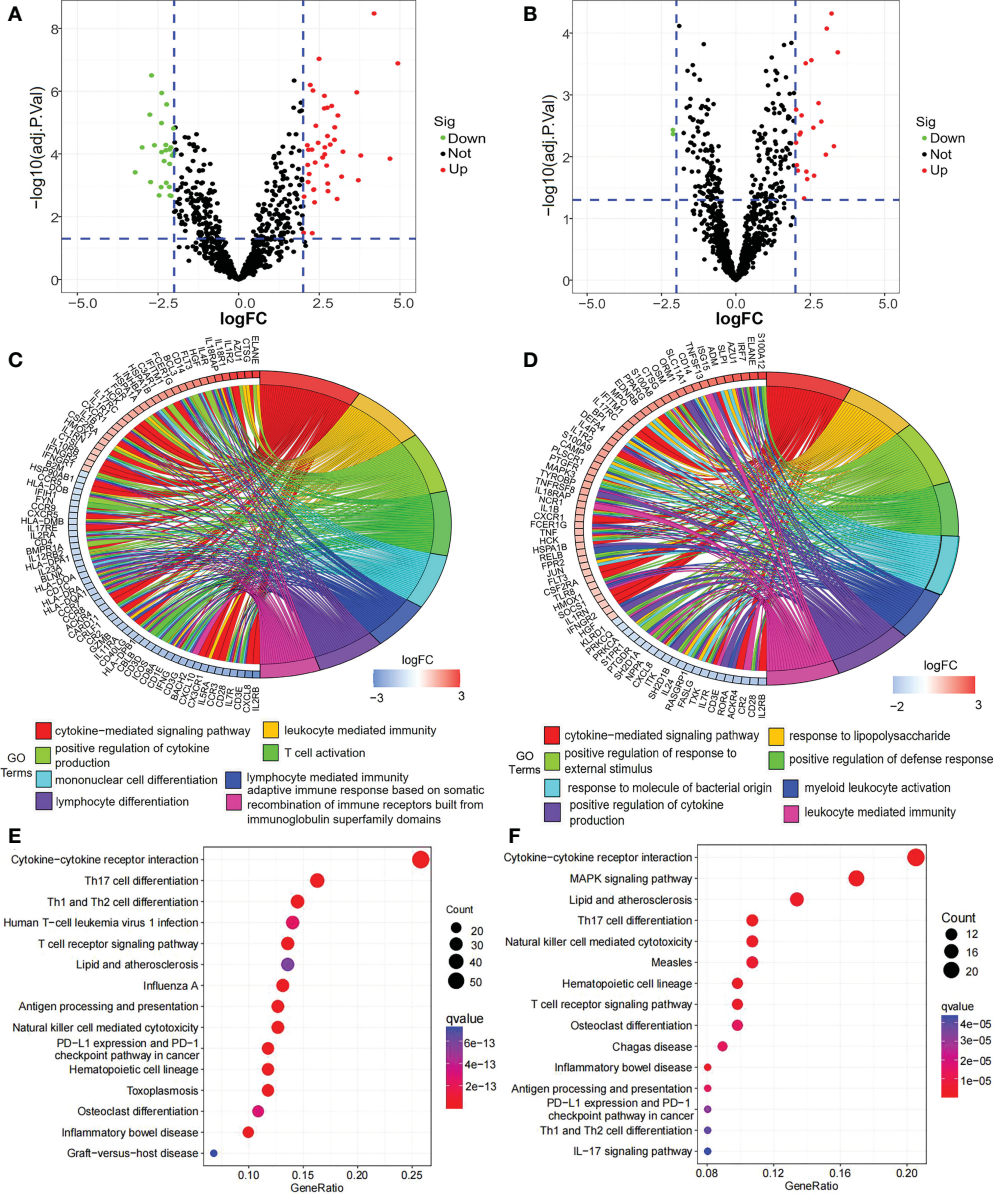
DEIGs and their enrichment analysis. **(A)** Volcano plot of DEIGs between the AIS A grade of SCI and the HC group. **(B)** Volcano plot of DEIGs between the AIS D grade of SCI and the HC group. **(C)** GO functional enrichment analysis of DEIGs between the AIS A grade of SCI and the HC group. **(D)** GO functional enrichment analysis of DEIGs between the AIS D grade of SCI and the HC group. **(E)** KEGG enrichment analysis of DEIGs between the AIS A grade of SCI and the HC group. **(F)** KEGG enrichment analysis of DEIGs between the AIS D grade of SCI and the HC group. SCI, spinal cord injury; HC, healthy uninjured control; DEIGs, differentially expressed immune-related genes; GO, Gene Ontology; KEGG, Kyoto Encyclopedia of Genes and Genomes; AIS, American Spinal Cord Injury Association Impairment Scale.

Subsequently, 3244, 638, 1907, and 2093 DEGs and 263, 68, 163, and 146 DEIGs of the AIS A, AIS B, AIS C and AIS D grades between the SCI and HC groups were obtained (**Supplemental Table 7**). Due to the small sample size for grades AIS B and AIS C, only grades AIS A and AIS D were analyzed (**Figures 5A, B**). GO functional enrichment analysis of DEIGs for the AIS A grade of SCI showed the main functions to be cytokine-mediated signal immunity, leukocyte activation, differentiation and proliferation, leukocyte adhesion and cell chemotaxis migration, antigen processing and presentation, and regulation of cytokine production, among others (**Figure 5C**; **Supplement Table 8**). KEGG enrichment analysis of DEIGs for the AIS A grade of SCI revealed that cytokine−cytokine receptor interactions, T-cell differentiation, antigen processing and presentation, natural killer cell-mediated cytotoxicity, vascular endothelial growth factor signaling, trans endothelial migration of leukocytes, axon guidance, neurotrophic factor signaling pathway, signaling pathway regulating stem cell pluripotency, sphingolipid signaling pathway, apoptosis and other signaling pathways were significantly altered (**Figure 5E**; **Supplement Table 9**). According to GO functional enrichment analysis of DEIGs for the AIS D grade of SCI, the main functions are cytokine-mediated signaling pathways, leukocyte activation, differentiation and proliferation, leukocyte chemotactic migration and adhesion, regulation of phagocytosis, cell killing, wound healing, and regulation of vascular-associated smooth muscle cell proliferation, among others (**Figure 5D**; **Supplement Table 10**). Cytokine−cytokine receptor interactions, MAPK signaling pathway, T-cell differentiation, T-cell receptor signaling pathway, antigen processing and presentation, lipids and atherosclerosis, neuroactive ligand−receptor interactions, sphingolipid signaling, complement and coagulation cascades, natural killer cell-mediated cytotoxicity and other signaling pathways were altered based on KEGG enrichment analysis of DEIGs for the AIS D grade of SCI (**Figure 5F**; **Supplement Table 11**).

**Figure 6:**
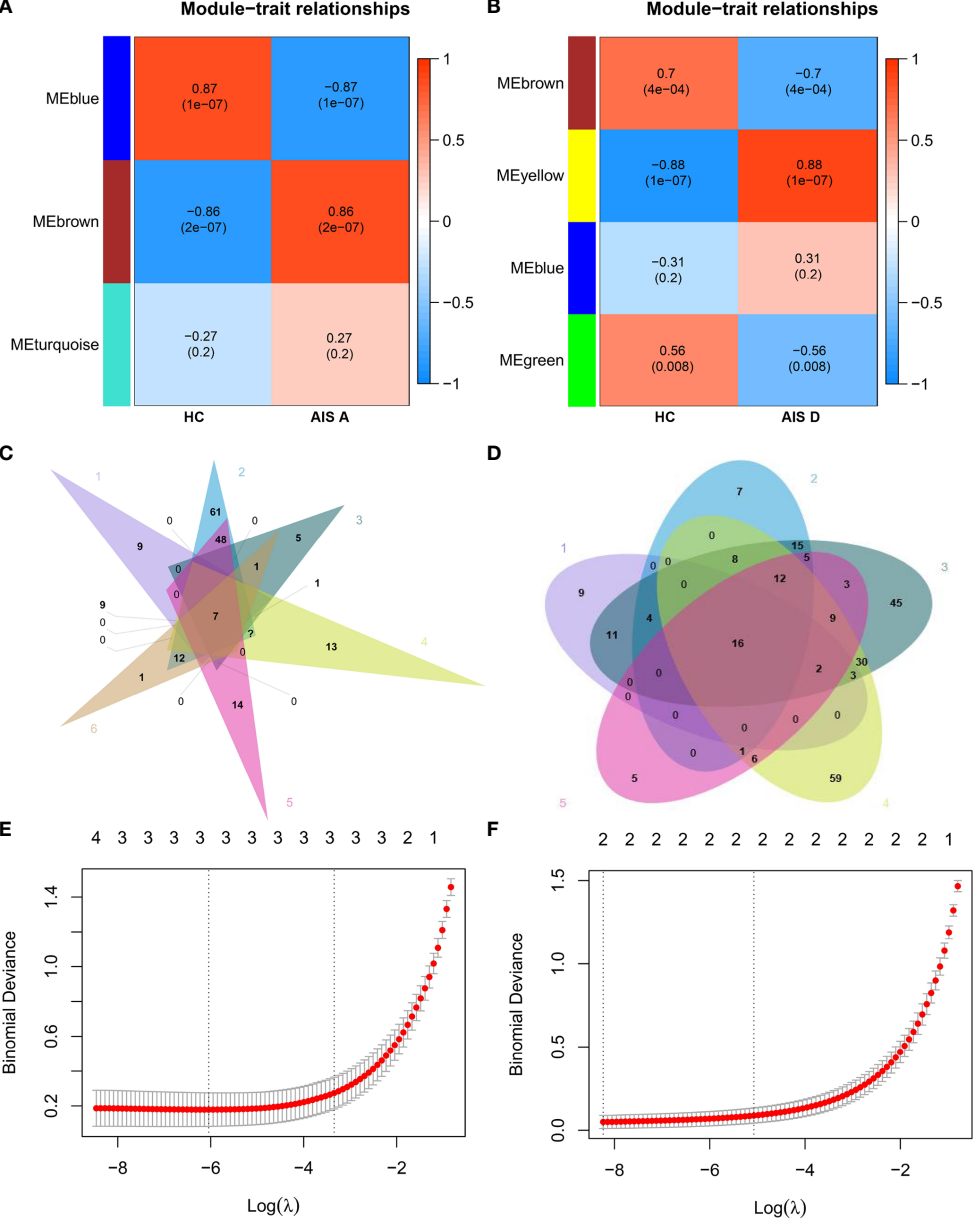
Identification of biomarkers of AIS A and AIS D grades of SCI. **(A)** Heatmap of correlations between module signature genes and clinical features of the AIS A grade of SCI. **(B)** Heatmap of correlations between module signature genes and clinical features of the AIS D grade of SCI. **(C)** Relatively specific DEIGs of the AIS A grade of SCI were obtained based on a Venn diagram. **(D)** Relatively specific DEIGs of the AIS D grade of SCI were obtained based on a Venn diagram. **(E)** LASSO logistic regression analysis of the AIS A grade of SCI. **(F)** LASSO regression analysis of the AIS D grade of SCI. SCI, spinal cord injury; HC, healthy uninjured control; AIS, American Spinal Cord Injury Association Impairment Scale; DEIGs, differentially expressed immune-related genes.

In addition, through WGCNA of immune-related genes of the AIS A grade of SCI, 3 different coexpression modules were obtained. Among them, the MEbrown module correlated highly positively with the AIS A grade of SCI; 87 shared immune-related genes of this module and 56 hub genes among the shared genes were obtained (**Figure 6A**). Then, we intersected the 56 hub genes with the DEIGs between the TC group and HC group and the DEIGs of the AIS A, AIS B, AIS C, and AIS D grades between the SCI and HC groups, and 12 immune-related genes that were only intersected among the DEIGs between the AIS D grade of the SCI group and HC group and hub genes were obtained (**Figure 6C**). Three important characteristic genes, GDF11, HSPA1L, and TNFRSF25, were revealed by analyzing expression of these 12 genes in the samples using LASSO logistic regression (**Figure 6E**).

Through WGCNA of the immune-related genes of the AIS D grade of SCI, 4 different gene coexpression modules were obtained. Among them, the MEbrown module correlated highly positively with the AIS D grade of SCI; 105 shared immune-related genes of this module and 58 hub genes among the shared genes were obtained (**Figure 6B**). We intersected these 58 hub genes with the DEIGs between the TC group and HC group and the DEIGs of the AIS B, AIS C and AIS D grades between the SCI and HC groups, and 6 immune-related genes that only intersected among the DEIGs between the AIS D grade of the SCI and HC groups were obtained (**Figure 6D**). Two important characteristic genes, CMTM2 and PRKCA, were identified by analyzing expression of these 6 genes in the samples using LASSO logistic regression (**Figure 6F**).

**Figure 7:**
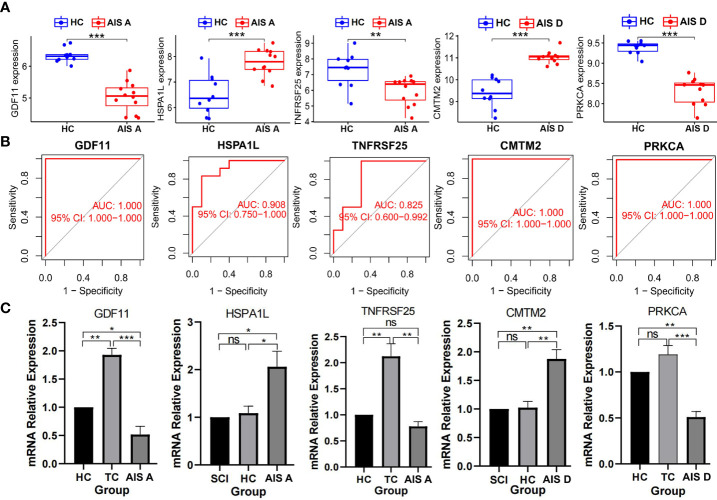
Detection of biomarkers of the AIS A and AIS D grades of SCI. **(A)** Expression of biomarkers between different grades of SCI and HC groups in the dataset. **(B)** Validation of the diagnostic value of biomarkers. **(C)** Expression levels of biomarkers detected by qPCR. Values are expressed as the means ± SEM. n = 3 for each group. Student’s t-test was used. ***P < 0.001; **P < 0.01; *P < 0.05; ns, no significance. A P value < 0.05 was regarded as statistically significant. SCI, spinal cord injury; HC, healthy uninjured control; TC, trauma control with noncentral nervous system injury; AIS, American Spinal Cord Injury Association Impairment Scale; AUC, area under the curve; qPCR, quantitative polymerase chain reaction.

Furthermore, expression of these characteristic biomarkers was detected in a dataset composed of the corresponding disease class and healthy group. GDF11 and TNFRSF25 expression was downregulated in SCI AIS A grade, whereas HSPA1L expression was significantly upregulated. PRKCA expression of was significantly downregulated in the AIS D grade of SCI, but that of CMTM2 was significantly upregulated (**Figure 7A**). The AUC values of these characteristic biomarkers in the dataset composed of the corresponding disease grade and control group were calculated: the AUC values of GDF11, HSPA1L, and TNFRSF25 in the AIS A grade of SCI were 1.000, 0.908, and 0.825, respectively. The AUC values of PRKCA and CMTM2 in the AIS D grade of SCI were both 1.000 (**Figure 7B**). Similarly, we used a qPCR assay to detect expression of the biomarkers in the HC group, TC group, and each grade of SCI and found that GDF11 showed low expression in the AIS A grade of SCI, while HSPA1L showed high expression in the AIS A grade of SCI. However, the expression of TNFRSF25 was not significantly elevated in the AIS A grade of SCI. PRKCA showed low expression in AIS D grade SCI, while CMTM2 showed high expression in the AIS D grade of SCI (**Figure 7C**).

**Figure 8:**
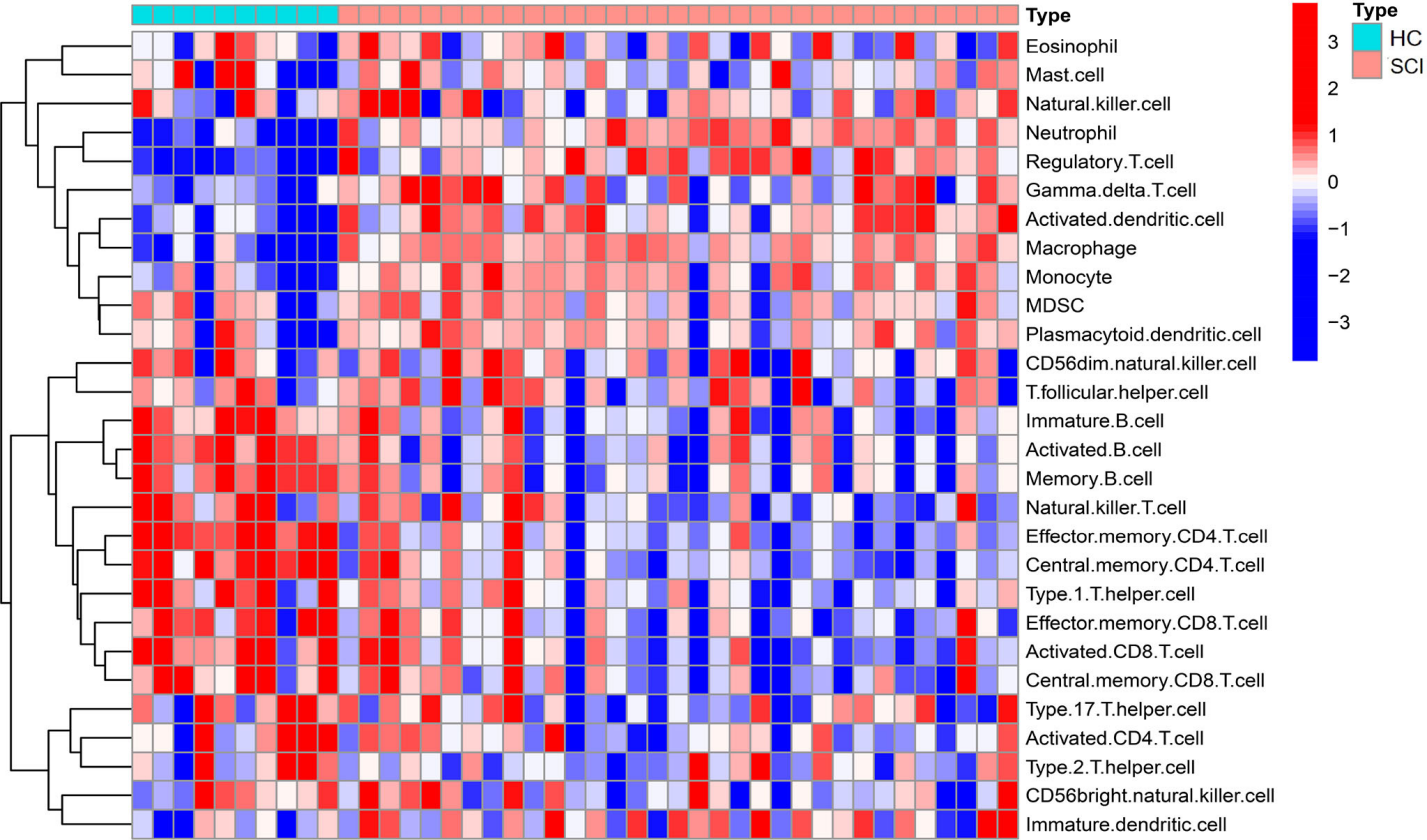
Heatmap of ssGSEA of immune cells in the SCI and HC groups. SCI, spinal cord injury; HC, healthy uninjured control. ssGSEA, single-sample gene set enrichment analysis.

**Figure 9:**
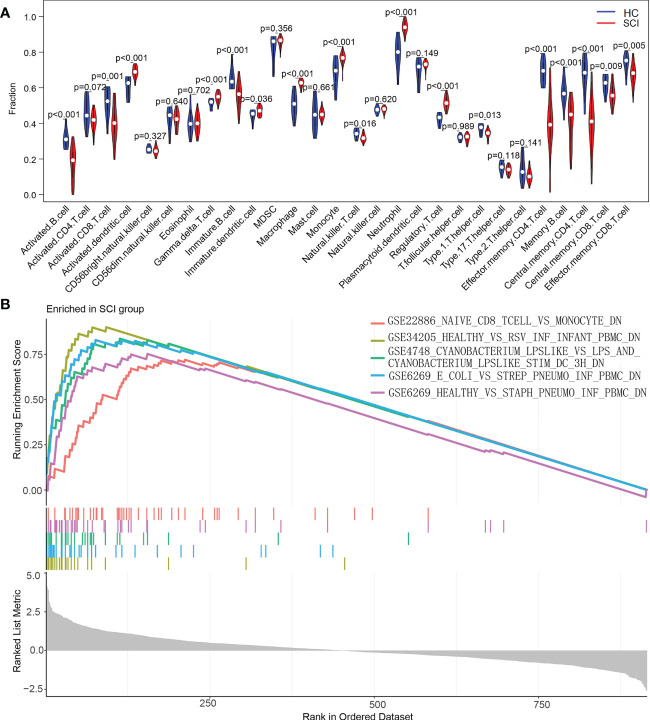
Analysis of the immune landscape associated with SCI. **(A)** Violin plot of differences in the content of immune cells between the SCI and HC groups analyzed using the ssGSEA algorithm. **(B)** Enrichment map of the GSEA Immunologic Signature Database of the SCI group. SCI, spinal cord injury; HC, healthy uninjured control. ssGSEA, single-sample gene set enrichment analysis; GSEA, gene set enrichment analysis.

Additionally, the content changes of immune cells in the blood of SCI patients were analyzed using ssGSEA. Compared with the HC group, the SCI group had significantly increased numbers of activated dendritic cells, gamma delta T cells, immature dendritic cells, macrophages, monocytes, neutrophils, and regulatory T cells. Conversely, activated B cells, activated CD8 T cells, immature B cells, type 1 T helper cells, effector memory CD4 T cells, memory B cells, central memory CD4 T cells, central memory CD8 T cells, effector memory CD8 T cells and natural killer T cells were decreased in this group (**Figures 8**, **9A**).

To explore the underlying mechanisms of immune function in SCI, the immune signature gene set from the MSigDB database was used as a reference for GSEA. A total of 120 gene sets were significantly enriched (|normalized enrichment score (NES)|>1; adjusted P value < 0.05). These gene sets were mainly enriched in CD4 T cells, naive CD8 T cells, monocytes, neutrophils, peripheral blood mononuclear cells (PBMCs) and dendritic cells (DCs). **Table 2** lists the top 15 enriched genomes. These results demonstrate the critical role that immune-related genes play in SCI (**Figure 9B**).

**Table 2:** Top 15 significant immunological signatures enriched by immunity in GSEA of SCI.

Gene set name	NES	adjusted p value
GSE10325_CD4_TCELL_VS_MYELOID_UP	-2.816036179	7.60E-08
GSE34205_HEALTHY_VS_RSV_INF_INFANT_PBMC_DN	2.692721828	7.60E-08
GSE4748_CYANOBACTERIUM_LPSLIKE_VS_LPS_AND_CYANOBACTERIUM_LPSLIKE_STIM_DC_3H_DN	2.562226281	7.60E-08
GSE6269_E_COLI_VS_STREP_PNEUMO_INF_PBMC_DN	2.630427235	7.60E-08
GSE6269_HEALTHY_VS_STAPH_PNEUMO_INF_PBMC_DN	2.514059264	7.60E-08
GSE10325_LUPUS_CD4_TCELL_VS_LUPUS_MYELOID_UP	-2.642310238	1.78E-07
GSE22886_NAIVE_CD8_TCELL_VS_MONOCYTE_DN	2.43149866	2.68E-07
GSE22886_NAIVE_CD4_TCELL_VS_MONOCYTE_DN	2.430116382	8.42E-07
GSE22886_NAIVE_CD8_TCELL_VS_MONOCYTE_UP	-2.572875931	1.11E-06
GSE11057_PBMC_VS_MEM_CD4_TCELL_DN	-2.526411013	1.67E-06
GSE10325_LUPUS_BCELL_VS_LUPUS_MYELOID_DN	2.426167896	2.10E-06
GSE22886_NAIVE_TCELL_VS_MONOCYTE_UP	-2.503352528	2.54E-06
GSE45739_UNSTIM_VS_ACD3_ACD28_STIM_WT_CD4_TCELL_DN	-2.559967388	3.92E-06
GSE22886_NAIVE_TCELL_VS_MONOCYTE_DN	2.350361117	6.14E-06
GSE22886_NAIVE_BCELL_VS_NEUTROPHIL_DN	2.350670653	7.89E-06

Adjusted p value < 0.0.5 was regarded as statistically significant.

**Figure 10:**
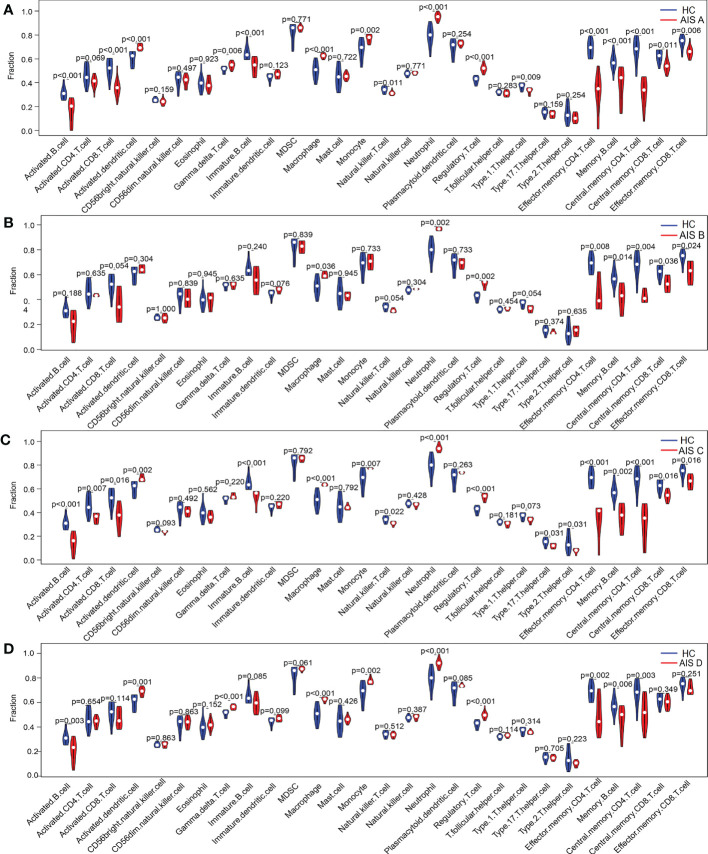
Differences in the content of immune cells between the SCI and HC groups analyzed using the ssGSEA algorithm. **(A–D)** Changes in immune cells in the blood of patients with SCI AIS A, AIS B, AIS C and AIS D grades using the ssGSEA algorithm. SCI, spinal cord injury; HC, healthy uninjured control; AIS, American Spinal Cord Injury Association Impairment Scale; ssGSEA, single-sample gene set enrichment analysis.

**Figure 11:**
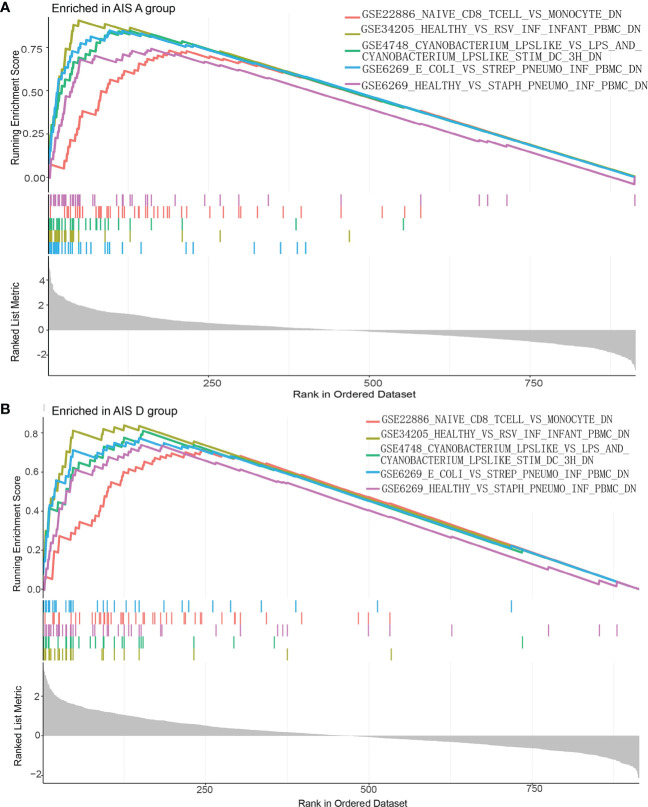
Enrichment map of the GSEA Immunologic Signature Database. **(A)** Enrichment map of the GSEA Immunologic Signature Database of the AIS A grade of the SCI group. **(B)** Enrichment map of the GSEA Immunologic Signature Database of the AIS D grade of the SCI group. SCI, spinal cord injury; HC, healthy uninjured control. GSEA, gene set enrichment analysis.

Similarly, we analyzed content changes of immune cells in the blood of patients with the AIS A, AIS B, AIS C and AIS D grades of SCI using the ssGSEA algorithm and found that activated dendritic cells, gamma delta T cells, macrophages, monocytes, neutrophils, and regulatory T cells were increased in all grades of SCI (**Figure 10**). To explore the underlying mechanisms of immune function in different disease grades of SCI, immune signature gene sets from the MSigDB database were used as a reference for GSEA of DEIGs. A total of 113 gene sets were significantly enriched (|NES|>1; adjusted P value < 0.05) in the blood of patients with SCI AIS A grade; 86 gene sets were significantly enriched (|NES| >1; adjusted P value < 0.05) in the blood of patients with SCI AIS D. The genomes in both SCI grades were mainly highly enriched in peripheral blood mononuclear cells (PBMCs), dendritic cells (DCs), monocytes, CD4 T cells, naive CD8 T cells and neutrophils. The top 15 enriched genomes of the AIS A grade and AIS D grade of SCI are listed in **Tables 3**, **4**, and the top 5 enriched genomes are listed in **Figure 11**, respectively. These results demonstrate the critical role that immune-related genes play in the development of SCI.

**Table 3:** Top 15 significant immunological signatures enriched by immunity in GSEA of the AISA grade of SCI.

Gene set name	NES	adjusted p value
GSE34205_HEALTHY_VS_RSV_INF_INFANT_PBMC_DN	2.435074907	1.06E-06
GSE4748_CYANOBACTERIUM_LPSLIKE_VS_LPS_AND_CYANOBACTERIUM_LPSLIKE_STIM_DC_3H_DN	2.430972786	1.59E-06
GSE6269_HEALTHY_VS_STAPH_PNEUMO_INF_PBMC_DN	2.42249961	1.93E-06
GSE6269_E_COLI_VS_STREP_PNEUMO_INF_PBMC_DN	2.397817025	2.38E-06
GSE22886_NAIVE_CD8_TCELL_VS_MONOCYTE_DN	2.350643814	3.16E-06
GSE10325_CD4_TCELL_VS_MYELOID_UP	-2.628736928	9.20E-06
GSE22886_NAIVE_BCELL_VS_MONOCYTE_DN	2.338277252	1.08E-05
GSE22886_NAIVE_TCELL_VS_MONOCYTE_UP	-2.591857115	1.08E-05
GSE22886_NAIVE_CD4_TCELL_VS_MONOCYTE_DN	2.328402481	1.19E-05
GSE6269_FLU_VS_STAPH_AUREUS_INF_PBMC_DN	2.348597677	1.45E-05
GSE29618_MONOCYTE_VS_MDC_UP	2.251360616	1.66E-05
GSE10325_LUPUS_BCELL_VS_LUPUS_MYELOID_DN	2.313828189	1.66E-05
GSE22886_NAIVE_CD4_TCELL_VS_MONOCYTE_UP	-2.554791656	2.69E-05
GSE25087_FETAL_VS_ADULT_TREG_DN	-2.49315635	2.72E-05
GSE10325_LUPUS_CD4_TCELL_VS_LUPUS_MYELOID_DN	2.277532261	3.25E-05

Adjusted p value < 0.0.5 was regarded as statistically significant.

**Table 4:** Top 15 significant immunological signatures enriched by immunity in GSEA of the AISD grade of SCI.

Gene set name	NES	adjusted p value
GSE10325_CD4_TCELL_VS_MYELOID_UP	-2.676687483	9.49E-08
GSE34205_HEALTHY_VS_RSV_INF_INFANT_PBMC_DN	2.676924382	9.49E-08
GSE4748_CYANOBACTERIUM_LPSLIKE_VS_LPS_AND_CYANOBACTERIUM_LPSLIKE_STIM_DC_3H_DN	2.599480907	9.49E-08
GSE6269_E_COLI_VS_STREP_PNEUMO_INF_PBMC_DN	2.7109932	9.49E-08
GSE6269_HEALTHY_VS_STAPH_PNEUMO_INF_PBMC_DN	2.525568489	1.01E-07
GSE22886_NAIVE_CD8_TCELL_VS_MONOCYTE_DN	2.507999372	1.75E-07
GSE22886_NAIVE_CD4_TCELL_VS_MONOCYTE_DN	2.498432399	1.75E-07
GSE10325_LUPUS_CD4_TCELL_VS_LUPUS_MYELOID_UP	-2.567069603	6.13E-07
GSE11057_PBMC_VS_MEM_CD4_TCELL_DN	-2.546067217	6.13E-07
GSE10325_LUPUS_BCELL_VS_LUPUS_MYELOID_DN	2.490119193	1.38E-06
GSE22886_NAIVE_TCELL_VS_MONOCYTE_DN	2.462969394	2.15E-06
GSE22886_NAIVE_CD8_TCELL_VS_MONOCYTE_UP	-2.530240915	3.62E-06
GSE22886_NAIVE_BCELL_VS_MONOCYTE_DN	2.38155792	4.21E-06
GSE45739_UNSTIM_VS_ACD3_ACD28_STIM_WT_CD4_TCELL_DN	-2.504928925	5.04E-06
GSE22886_NAIVE_TCELL_VS_MONOCYTE_UP	-2.537709297	5.04E-06

Adjusted p value < 0.0.5 was regarded as statistically significant.

**Figure 12:**
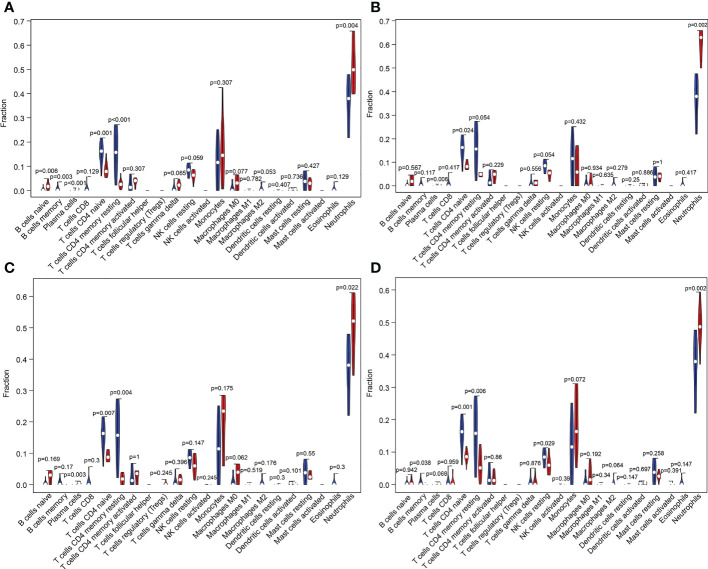
Violin plot of differences in the content of immune cells between different grades of SCI and the HC group analyzed using the CIBERSORT algorithm. **(A–D)** Changes in immune cells in the blood of patients with SCI AIS A, AIS B, AIS C and AIS D grades using the CIBERSORT algorithm. SCI, spinal cord injury; HC, healthy uninjured control; AIS, American Spinal Cord Injury Association Impairment Scale; CIBERSORT, Cell-type Identification by Estimating Relative Subsets of RNA Transcripts.

We also performed CIBERSORT algorithm analysis and found that naive B cells, plasma cells, monocytes, and neutrophils were significantly increased in grade AIS A compared with the HC group and that memory B cells and naive CD4 T cells were significantly decreased (**Figure 12A**). In grade AIS B, plasma cells and neutrophils were increased significantly, and naive CD4 T cells significantly decreased (**Figure 12B**). In grade AIS C, plasma cells and neutrophils were increased significantly, whereas CD4 naive T cells and resting memory CD4 T cells were decreased significantly (**Figure 12C**). In grade AIS D, neutrophils were increased significantly, and memory B cells, naive CD4 T cells, resting CD4 memory T cells and resting NK cells decreased significantly (**Figure 12D**).

**Figure 13:**
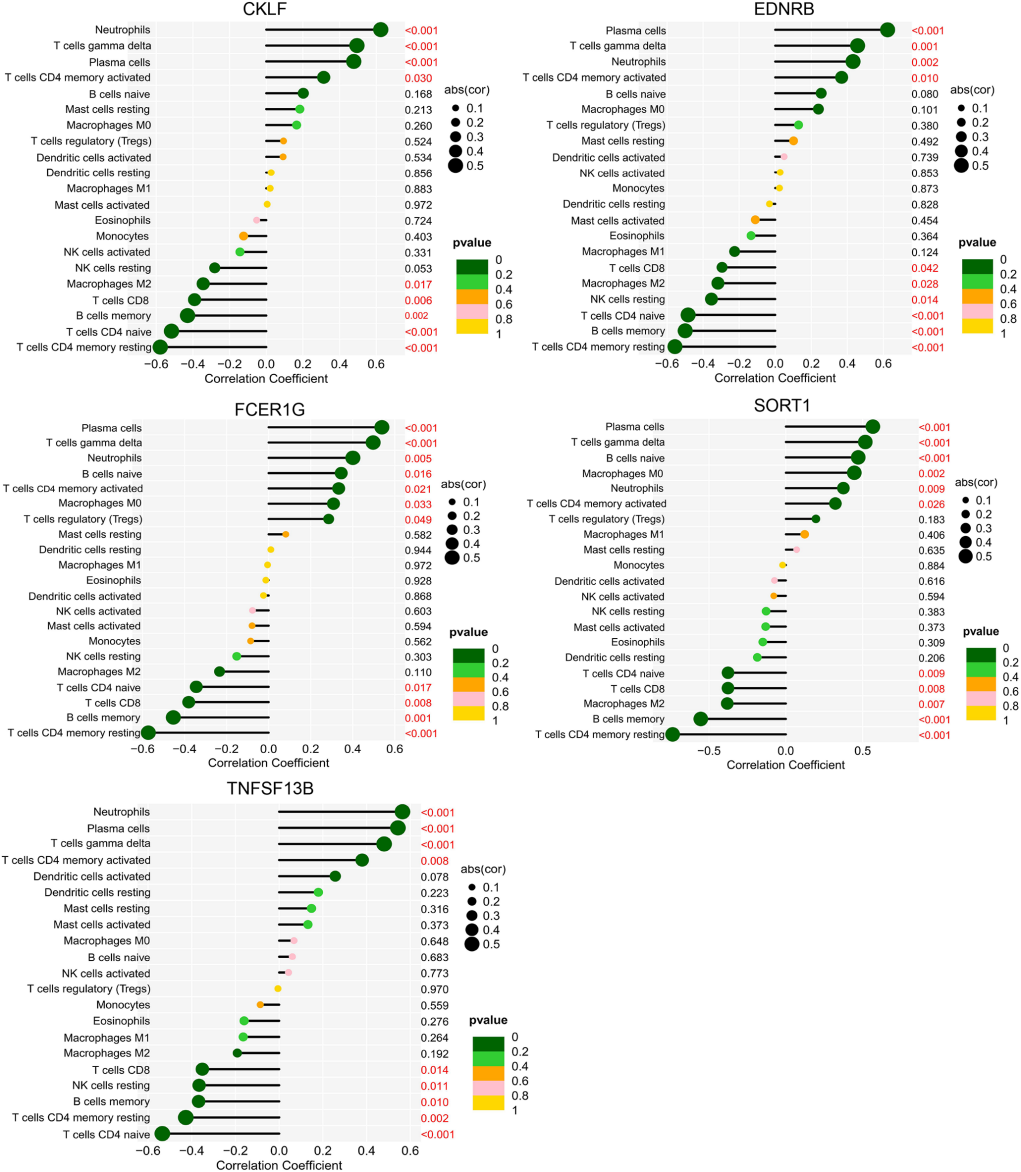
Correlation between biomarkers and immune cells in SCI. The ordinate on the left represents the proportion of immune cells, the ordinate on the right represents the P value of the correlation test, and the abscissa represents the correlation coefficient. The size of the circle represents the correlation coefficient, and the color of the circle represents the P value of the correlation test; a P value<0.05 was considered statistically significant. SCI, spinal cord injury.

Correlation analysis of biomarkers and immune cells showed that CKLF, EDNRB, SORT1, and TNFSF13B correlated positively with neutrophils, plasma cells, gamma delta T cells, and CD4 memory-activated T cells after SCI. In addition, FCER1G correlated positively with regulatory T cells (Tregs) and M0 macrophages. TNFSF13B also correlated positively with M0 macrophages and naive B cells (**Figure 13**).

**Figure 14:**
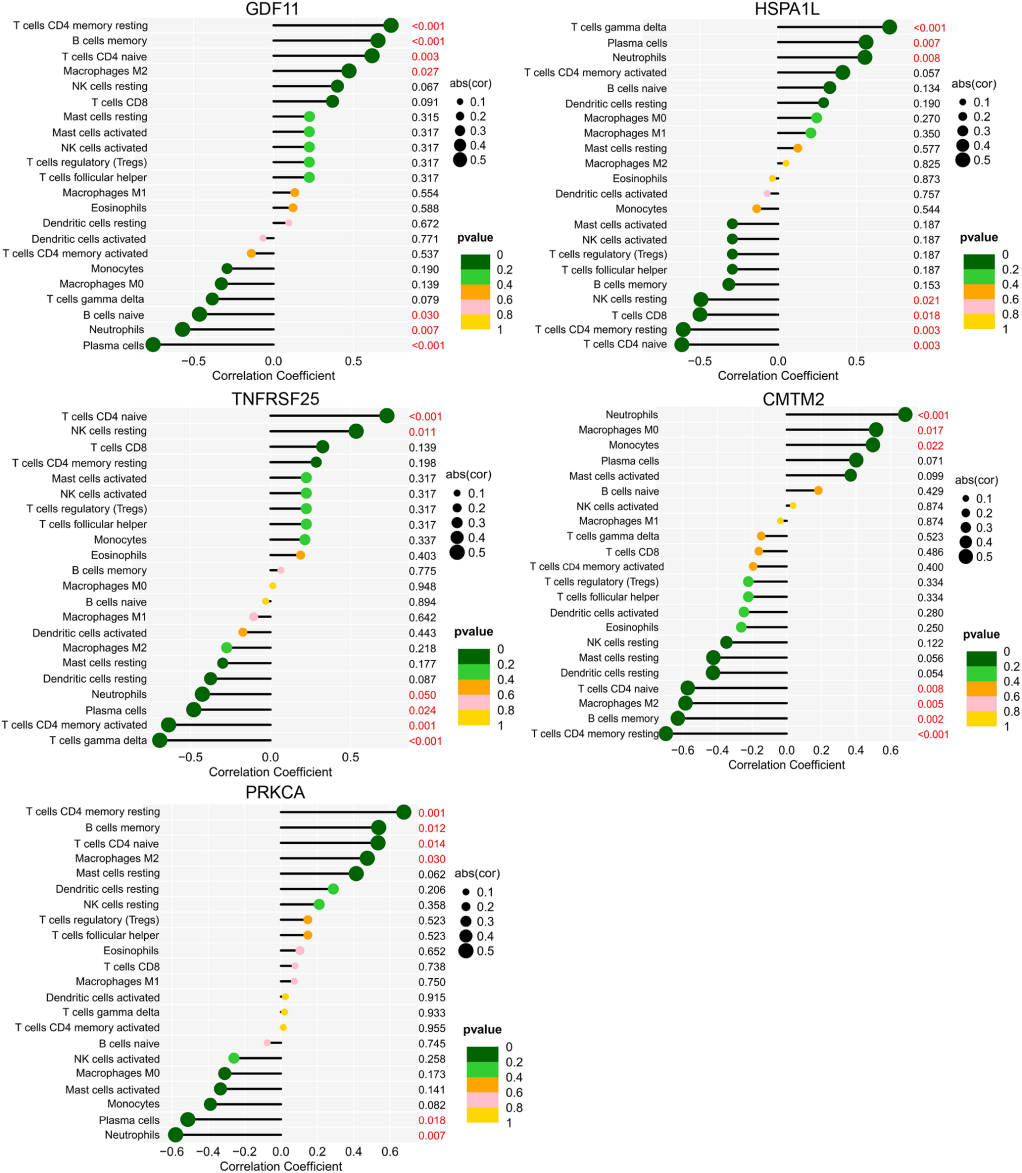
Correlation between biomarkers and immune cells of the AIS A and AIS D grades of SCI. The ordinate on the left represents the proportion of innate immune cells, the ordinate on the right represents the P value of the correlation test, and the abscissa represents the correlation coefficient. The size of the circle represents the correlation coefficient, and the color of the circle represents the P value of the correlation test; a P value<0.05 was considered statistically significant. SCI, Spinal cord injury; AIS, American Spinal Cord Injury Association Impairment Scale.

GDF11 correlated positively with resting memory CD4 T cells, memory B cells, naive CD4 T cells, and M2 macrophages and negatively with naive B cells, neutrophils, and plasma cells. HSPA1L correlated positively with gamma delta T cells, neutrophils, and plasma cells and negatively with resting NK cells, CD8 T cells, resting memory CD4 T cells, and naive CD4 T cells. TNFRSF25 correlated positively with CD4 naive T cells and resting NK cells and negatively with CD4 memory activated T cells, neutrophils, plasma cells, and gamma delta T cells. CMTM2 correlated positively with neutrophils, M0 macrophages, and monocytes and negatively with memory B cells, resting memory CD4 T cells, M2 macrophages, and naive CD4 T cells. PRKCA correlated positively with memory B cells, resting memory CD4 T cells, M2 macrophages, and naive CD4 T cells and negatively with neutrophils and plasma cells (**Figure 14**).

## 4 Discussion

In this study, DEGs and DEIGs in blood after SCI and characteristic immune biomarkers of SCI were obtained, as were DEGs and DEIGs of each grade of SCI and characteristic biomarkers of AIS A and AIS D grades of SCI, providing important help and research ideas for diagnosis of SCI and its severity. In addition, characteristics of immune cell changes in the blood of human SCI and patients with different grades of SCI were analyzed, and correlations between characteristic biomarkers and 28 kinds of immune cells in the blood were further studied. The findings contribute to a more comprehensive understanding of the changes in immune cells in blood after SCI.

At present, accurate assessment of the severity of SCI and the prognosis of SCI based on imaging examination and physical examination is limited. The use of serum biomarkers to assist in the diagnosis of SCI may be of great significance (34, 35). In this study, to obtain accurate results, WGCNA and LASSO regression analysis were used to determine diagnostic biomarkers of SCI and its different grades. We first discovered five important SCI characteristic biomarkers, CKLF, EDNRB, FCER1G, SORT1, and TNFSF13B; their AUC values were 0.918, 0.924, 0.937, 0.987, and 0.979, respectively, indicating that they have certain characteristics for SCI, with relatively high diagnostic sensitivity. Moreover, data analysis and qPCR results showed these biomarkers to be upregulated in the SCI group, which verified the accuracy of these biomarkers for SCI. Further analysis showed that the characteristic biomarkers of SCI AIS A grade were GDF11 and HSPA1L, with AUC values of 1.000 and 0.908, respectively; characteristic biomarkers of SCI AIS D grade were RKCA and CMTM2, with an AUC value of 1.000 for both.

In addition, data analysis and qPCR results showed these biomarkers to be downregulated or upregulated in the blood of AIS A grade and AIS D grade patients with SCI. Therefore, these characteristic biomarkers of two different grades may have high diagnostic sensitivity and specificity for corresponding grades of SCI, which may be helpful for diagnosis and treatment of the corresponding SCI degree.

Furthermore, we found that the numbers of activated dendritic cells and immature dendritic cells in the blood of SCI patients were significantly increased compared with those in the blood of HCs, indicating that dendritic cells become activated and differentiated after SCI. Macrophages, monocytes, neutrophils, gamma delta T cells, and regulatory T cells can all penetrate the SCI area through the broken blood−brain barrier and play an important role in the formation of the local immune microenvironment (11–17). The increase in these cells in blood also indicates that SCI causes an increase in innate immune cells and adaptive immune activity in the peripheral blood. KEGG analysis of DEIGs in SCI revealed changes in cytokine−cytokine receptor interactions, T-cell and B-related signaling pathways, antigen processing and presentation, chemokine signaling pathways, vascular endothelial growth factor signaling pathways, axon guidance, leukocyte trans endothelial migration and other signaling pathways, indicating that the injured spinal cord tissue releases inflammatory chemokines, playing a role in promoting penetration of blood immune cells into the injured area through the blood-brain barrier. Overall, these immune cells may play a role in the process of axonal growth and angiogenesis.

We also found that activated dendritic cells, gamma delta T cells, macrophages, monocytes, neutrophils, and regulatory T cells were increased in the blood of SCI patients with all grades, indicating that these immune cells play a more important role after SCI. KEGG enrichment analysis of AIS A grade DEIGs revealed changes in antigen processing and presentation, vascular endothelial growth factor signaling, leukocyte trans endothelial migration, axon guidance, neurotrophic factor signaling, regulation of stem cell pluripotency signaling pathway, sphingolipid signaling pathway and other signaling pathways. Hence, immune cells in the blood after SCI may be induced by inflammatory chemokines in the injured area and migrate toward the injured area. It also indicates that immune cells in the blood may play a role in the phagocytosis and clearance of myelin debris, axonal growth, and angiogenesis in SCI. KEGG enrichment analysis of AIS D grade DEIGs also revealed changes in antigen processing and presentation, neuroactive ligand−receptor interactions, and sphingolipid signaling pathways. However, there was no change in axon guidance, the neurotrophic factor signaling pathway, or the vascular endothelial growth factor signaling pathway, which may indicate that the severity of SCI AIS D grade is relatively mild.

CKLF is a chemokine, and isoform 1 is a potent chemokine for neutrophils (36, 37). In this study, expression of CKLF and the content of neutrophils were increased, with a positive correlation, indicating that CKLF may play a role in the process of neutropenia. EDNRB is also closely related to neutrophils (38). In our study, expression of EDNRB in SCI was significantly upregulated and correlated positively with neutrophils, indicating that EDNRB may also play a role in neutropenia. In addition, SORT1 and TNFSF13B correlated positively with neutrophils, possibly playing a role in the increase in neutrophil count. In our study, neutrophils and regulatory T cells were elevated in the blood at several hours after SCI, and expression levels of CKLF, EDNRB, SORT1, TNFSF13B, and FCER1G were all upregulated. Therefore, detecting changes in CKLF-neutrophils, EDNRB-neutrophils, SORT1-neutrophils, TNFSF13B-neutrophils, EDNRB-regulatory T cells, FCER1G-regulatory T cells, and SORT1-regulatory T cells in blood within hours after SCI may be helpful for diagnosing SCI from the perspective of blood examination.

The biomarker of AIS A grade SCI GDF11, transforming growth factor 11, acts by binding to TGF-β type I and II receptors. In cerebral infarction, it can improve cerebral nerve function by promoting angiogenesis in the peripheral area of the cerebral cortex (39); in myocardial infarction, it promotes the proliferation and angiogenesis of endothelial progenitor cells (40). In SCI, GDF11 protects the injured spinal cord by inhibiting pyroptosis and necroptosis through TFE3-mediated enhancement of autophagy (41). GDF11 also promotes nerve regeneration after sciatic nerve injury in adult rats by promoting axonal growth and inhibiting neuronal apoptosis (42). However, there is no research on the specific mechanism of GDF11 in the immune response to SCI. TNFRSF25, tumor necrosis factor receptor family 25, is mainly expressed in peripheral blood leukocytes and immune organs (43). As a T-cell costimulator, it functions by activating a transcription factor (NF-kB) or the PI3K/Akt axis (44).

Among the characteristic biomarkers of SCI AIS D grade, CMTM2 was correlated positively with macrophages and activated dendritic cells. PRKCA correlated negatively with type 2 T helper cells and activated CD4 T cells. CMTM2, a member of the CMTM family and characteristic biomarker of SCI AIS D, is widely expressed in the immune system, is involved in T-cell and B-cell activation and is closely related to autoimmune diseases such as antiphospholipid syndrome (45, 46). However, CMTM2 has not been studied in SCI tissue and blood. PRKCA is a subtype of the PKC family that participates in processes such as cell proliferation, differentiation, apoptosis, cell adhesion and migration, cell differentiation, tumorigenesis, and inflammation by activating signaling cascades (47–49). Nevertheless, the role of PRKCA in SCI has not yet been elucidated.

In our study, GDF11 was downregulated hours after SCI and correlated positively with resting memory CD4 T cells, memory B cells, naive CD4 T cells, and M2 macrophages and negatively with naive B cells, neutrophils, and plasma cells. Expression of HSPA1L was upregulated and correlated positively with gamma delta T cells, neutrophils, and plasma cells and negatively with resting NK cells, CD8 T cells, resting memory CD4 T cells, and naive CD4 T cells. Therefore, our findings suggest that detecting changes in GDF11 and HSPA1L expression in the blood of SCI patients as well as changes in the abovementioned related immune cells within a few to 48 hours after SCI may be helpful for diagnosis of the AIS A grade of SCI.

In addition, expression of CMTM2 was upregulated a few hours after SCI, and it correlated positively with neutrophils, M0 macrophages, and monocytes and negatively with memory B cells, resting memory CD4 T cells, M2 macrophages, and naive CD4 T cells. PRKCA expression was downregulated, correlating positively with memory B cells, resting memory CD4 T cells, M2 macrophages, and naive CD4 T cells and negatively with neutrophils and plasma cells. Therefore, detection of the gene expression levels of CMTM2 and PRKCA and changes in related immune cells in the blood of patients from a few hours to 48 hours after SCI may be helpful for diagnosing the AIS D level in SCI.

In conclusion, our study not only describes changes in immune cells in the blood of human SCI and its different grades but also identifies diagnostic biomarkers for SCI and different grades of SCI. Detecting expression of these diagnostic biomarkers and changes in related immune cells may be helpful for predicting the severity of SCI. However, the sample size of this study was relatively small, as this was the only dataset with statistically significant human SCI blood gene sequencing that we could retrieve from the database. In addition, this study was only carried out from the perspective of the gene transcriptome and only bioinformatics methods were applied for data analysis. And only simple verification experiments were performed, therefore, experimental verification with large sample size will be carried out in the future.

## Data availability statement

The datasets presented in this study can be found in online repositories. The names of the repository/repositories and accession number(s) can be found below: https://www.ncbi.nlm.nih.gov /, GSE151371.

## Ethics statement

Ethical review and approval was not required for the study on human participants in accordance with the local legislation and institutional requirements. Written informed consent for participation was not required for this study in accordance with the national legislation and the institutional requirements.

## Author contributions

JL: Methodology, Formal analysis, Investigation, Writing-original draft. XL: Methodology, Investigation, Writing-original draft. JMW: Methodology, Investigation, Writing-original draft. FW: Formal analysis, Investigation, Writing-review and editing. ZYZ: Methodology, Writing-review and editing. TT: Methodology, Writing-review and editing. JW: Writing-review and editing. ZZ: Conceptualization, Project administration, Funding acquisition. MG: Conceptualization, Data curation, Writing- review and editing, Revision, Project administration. SL: Conceptualization, Resources, Supervision, Project administration, Funding acquisition. All authors contributed to the article and approved the submitted version.
